# Social determinants of antibiotic misuse: a qualitative study of community members in Haryana, India

**DOI:** 10.1186/s12889-017-4261-4

**Published:** 2017-04-19

**Authors:** Anna K. Barker, Kelli Brown, Muneeb Ahsan, Sharmila Sengupta, Nasia Safdar

**Affiliations:** 1 Department of Population Health Sciences, University of Wisconsin, School of Medicine and Public Health, Madison, WI USA; 2 Medanta Institute of Education and Research, Medanta the Medicity Hospital, Gurgaon, Haryana India; 3 Department of Clinical Microbiology & Infection Control, Medanta the Medicity Hospital, Gurgaon, Haryana India; 4 Department of Medicine, University of Wisconsin, School of Medicine and Public Health, Madison, WI USA; 5 William S. Middleton Memorial Veterans Affairs Hospital, Madison, WI USA

**Keywords:** Antibiotic resistance, Social determinants, Global health, India, Rural health, Qualitative research

## Abstract

**Background:**

Antibiotic resistance is a global public health crisis. In India alone, multi-drug resistant organisms are responsible for over 58,000 infant deaths each year. A major driver of drug resistance is antibiotic misuse, which is a pervasive phenomenon worldwide. Due to a shortage of trained doctors, access to licensed allopathic doctors is limited in India’s villages. Pharmacists and unlicensed medical providers are commonly the primary sources of healthcare. Patients themselves are also key participants in the decision to treat an illness with antibiotics. Thus, better understanding of the patient-provider interactions that may contribute to patients’ inappropriate use of antibiotics is critical to reducing these practices in urban and rural Indian villages.

**Methods:**

We conducted a qualitative study of the social determinants of antibiotic use among twenty community members in Haryana, India. Semi-structured interview questions focused on two domains: typical antibiotic use and the motivation behind these practices. A cross-sectional pilot survey investigated the same twenty participants’ understanding and usage of antibiotics. Interview and open-ended survey responses were translated, transcribed, and coded for themes.

**Results:**

Antibiotics and the implications of their misuse were poorly understood by study participants. No participant was able to correctly define the term antibiotics. Participants with limited access to an allopathic doctor, either for logistic or economic reasons, were more likely to purchase medications directly from a pharmacy without a prescription. Low income participants were also more likely to prematurely stop antibiotics after symptoms subsided. Regardless of income, participants were more likely to seek an allopathic doctor for their children than for themselves.

**Conclusions:**

The prevalent misuse of antibiotics among these community members reinforces the importance of conducting research to develop effective strategies for stemming the tide of antibiotic resistance in India’s villages.

## Background

Antibiotic resistance is a global public health crisis. Reducing the incidence of drug resistant infections is crucial and is a top priority of the World Health Organization (WHO), pharmaceutical industry, and many national health agendas [1–6]. A major driver of drug resistance is antibiotic misuse, which is pervasive worldwide. Misuse contributes to the spread of multidrug resistant organisms (MDROs; 2). Infections caused by MDROs result in longer hospital stays and increased morbidity and mortality [7]. At least 23,000 people die from MDRO infections in the United States each year [2]. Yet, the burden in low and middle income countries is estimated to be three times higher per patient-hospitalization day [8]. In India, MDROs are responsible for over 58,000 deaths each year in the neonate population alone [6].

To date, much of the research into the practices, prevalence, and patient perspectives of antibiotic misuse have been conducted in the United States and Europe [9–15]. Studies of patients in these settings often focus on their interaction with doctors or advanced practice providers, who control access to antibiotics through written prescriptions. Due to a shortage of trained doctors, access to licensed allopathic doctors is limited in India’s villages. Thus, pharmacists and unlicensed rural medical providers (RMPs) are commonly the primary sources of healthcare [16–18].

The role of pharmacists as first-line healthcare providers has made it difficult to implement strict regulatory mechanisms to restrict over the counter dispensing of antibiotics. As a result, several leading Indian non-governmental healthcare societies collaborated with the WHO to release the 2012 Chennai Declaration, a five year plan focused on the practical implementation of antibiotic policies in India [19]. In 2014, the Indian government subsequently enacted Schedule H1, which increases dispensing restrictions nationwide for several antibiotic classes [20]. In recent years, policy makers have played an important role in limiting inappropriate antibiotic dispensing through top-down approaches.

Patients themselves are also key participants in the decision to treat an illness with antibiotics. Thus, better understanding of the patient-provider interactions that may contribute to patients’ inappropriate use of antibiotics is critical to reducing these practices in urban and rural Indian villages. Studies investigating patients and the patient-provider relationship in the context of antibiotic misuse have been conducted primarily in regions of India with more developed healthcare infrastructure [21–23]. Access to healthcare for the nearly 70% of India’s population living in villages is a greater challenge than in cities [24, 25]. Understanding the patient-provider relationship and its role in antibiotic use is especially crucial in understudied areas with limited healthcare access. We hypothesized that patients with less healthcare access, health knowledge, and lower incomes would be less likely to seek healthcare from an allopathic doctor, and that this would lead to greater antibiotic misuse due to the ease of purchasing antibiotics over the counter. Thus, we conducted a qualitative study to assess the social determinants of antibiotic use among community members in villages in the northern state of Haryana, India.

## Methods

We performed a qualitative study of the social determinants of antibiotic use among community members in Haryana, India, to investigate how healthcare access, health knowledge, and income impacts patients’ antibiotic use practices. The study included semi-structured interviews and a cross-sectional survey. Interview and survey data from twenty participants were collected in July and August 2015. This study was nested within a larger mixed-methods study of antibiotic misuse and dispensing among community members and healthcare professionals.

### Study population

Twenty participants were recruited in villages in the northern state of Haryana, India. Haryana borders the National Capital Territory of Delhi and has experienced rapid population growth over the past two decades due to high rates of immigration from other parts of India. The state’s poverty rate (12.50%), literacy rate (75.55%), and proportion of the population with a graduate education (12.39%) are improved compared to India as a whole (21.80, 72.98, and 9.51% respectively), while the under-eighteen percentage is similar (Haryana, 35.97%; India, 36.68%), and the level of homelessness is worse than the national average (0.20 vs. 0.15%) [22, 23].

The Indian village designation is based on population, not urbanicity, and ranges in size from less than 500 to over 10,000 people. Mid-sized villages, of population 2000–5000, account for nearly one-quarter of India’s overall population (23.8%) [24]. Three rural villages, Sikandarpur Badha, Bhirawati, and Silani, and two urban villages, Kadipur and Pratap Nagar, were selected for this study by convenience sampling. A pre-identified advocate in each village was utilized to increase support for the project within the communities.

A sample size of twenty interviews was finalized based on theoretical saturation. Theoretical saturation is a standard method for determining sample size in qualitative studies [26–28]. Using this method, saturation was reached and interviewing was stopped when interview responses became repetitive, such that little new material or analytic themes were gleaned from additional interviews. Fifteen community members were recruited by convenience sampling at local pharmacies, with the approval of the pharmacist or shop owner. To reach theoretical saturation, five additional people were recruited from nearby locations and roadside food stalls.

All English or Hindi speaking adults living in the state of Haryana who had purchased medicine from a pharmacy in the past three months were eligible to participate in the study. Exclusion criteria included being younger than eighteen, although ultimately no children sought enrollment in our study. Community members with formal degree-granting medical, pharmacy, or allied-health training, (e.g., Bachelor of Medicine and Surgery degree [MBBS] or Diploma in Pharmacy [D. Pharm]) were also excluded, because this group is not expected to be representative of the typical villager in terms of antibiotic knowledge and use practices.

### Study definitions

**Fig. 1:**
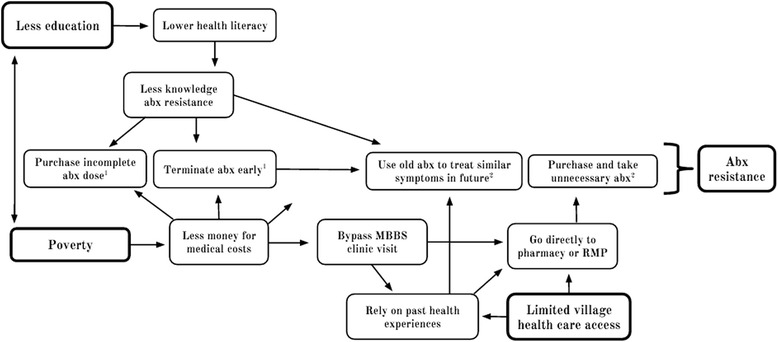
Hypothesized relationships between education, income, healthcare access, and antibiotic resistance. The four central outcomes, purchasing an incomplete antibiotic dose, terminating antibiotics early, using old antibiotics to treat similar symptoms in the future, and purchasing and taking unnecessary antibiotics, were all predicted to be associated with an increase in antibiotic resistance (far right). abx = antibiotics. ^1^Classified as shortened antibiotic courses; ^2^Classified as antibiotic overuse

In this study we used the terms antibiotic misuse and inappropriate use interchangeably. These terms were used to describe the following practices: 1) purchasing an incomplete dose of medication that is less than what was prescribed by an allopathic doctor, 2) stopping antibiotic treatment before all the doses are completed, 3) taking old antibiotics that were previously purchased to treat another illness, or 4) purchasing and taking any antibiotic without a prescription from an allopathic doctor. We categorized the first two practices as shortened antibiotic courses and the second two as antibiotic overuse (Fig. 1).

There are several potential meanings for the term “doctor” in India. To mitigate ambiguity, we refer to medical practitioners throughout this paper using designations based on the provider’s type and level of training. Allopathic medicine is a 5.5-years undergraduate program in India, leading to an MBBS. Doctors with an MBBS degree are the only providers who can legally prescribe antibiotics. Alternative medicine programs also require 5.5 years of undergraduate training, resulting in Bachelors degrees in Ayurvedic Medicine and Surgery (BAMS), Homoeopathic Medicine and Surgery (BHMS), Unani Medicine and Surgery (BUMS), etc. BAMS, BHMS, and BUMS doctors are fully trained in their respective alternative medical fields, but receive no allopathic medical training and are not licensed to prescribe or dispense antibiotics. Unlicensed rural medical practitioners (RMPs) are a third group that provides healthcare. These practitioners do not have formal medical training and cannot legally provide medical care, write antibiotic prescriptions, or dispense medications. Many RMPs worked previously as an assistant in the clinic of a qualified allopathic or alternative medicine doctor and their medical knowledge is gained by experience. In the absence of qualified MBBS practitioners, RMPs act as a primary source of healthcare in villages [29].

Pharmacies, called medical stores locally, are another first point of healthcare access. Pharmacist training is highly variable in India and dispensing of medications by employees with no formal pharmacy training is common (i.e. without a Diploma in Pharmacy or a Bachelors in Pharmacy) [30]. Throughout this paper we use the term pharmacist to refer to anyone dispensing medications at a medical store unsupervised, regardless of their clinical training.

### Interviews

We conducted twenty semi-structured interviews to investigate antibiotic misuse among village community members. Figure 1 provided the rationale for the initial interview guide. This guide was refined twice during the study based on participant responses. Interview questions focused on two domains: typical antibiotic use and the motivation behind these practices. The term antibiotic was defined and common examples of this medication class were provided to all participants. To investigate typical antibiotic use we assessed participants’ preferred sources of healthcare in mild and serious illnesses, prescription usage when purchasing antibiotics, sharing of unused medication, and typical antibiotic course durations. To examine underlying motivations for these practices, we asked participants to discuss their economic situation, any prior education or training in health, and why they prefer certain types of healthcare. Sample questions include: What are your first and second preferred options for obtaining healthcare when you have a mild illness and why? and How important to you is the cost of a medication, when deciding to take less antibiotics than a doctor recommends?

All interviews were conducted by English-Hindi bilingual members of the study team in the language that the participant preferred. English and Hindi are the two official national languages of India. Hindi is the only official local language in Haryana and English fluency among village populations on specialized health topics was rare. Thus, most interviews were conducted in Hindi, with real-time translation into English. Interviews were audio recorded and most lasted ten to fifteen minutes. Transcription of the interviews for data analysis was conducted first in Hindi, with subsequent translation into English.

### Analysis

Data analysis began during fieldwork, in order to identify reoccurring themes across participant responses. These initial findings motivated iterative re-workings of the interview guide, to delve deeper into the themes deemed most important or novel. Once data collection was complete, interview transcripts and open-ended survey data were analyzed with Nvivo software (Version 10.2, QSR International). Responses were coded for themes focusing on two domains: typical antibiotic use and the motivation behind these practices. Codes reflected the questions outlined in the interview guide. Two investigators independently coded the data and reviewed these analyses together.

### Questionnaire

A Knowledge, Attitude, and Practices questionnaire was developed to assess health literacy, typical antibiotic use, and antibiotic knowledge. This questionnaire includes both open- and closed-ended questions and was adapted from other Knowledge, Attitude, and Practices surveys of antibiotic use in low resources settings [31–34]. The questionnaire has three sections: demographic information (occupation, education, income, etc.), antibiotic consumption (for the participant and their dependents), and antibiotic knowledge. It was finalized after review by an international (United States/India) study team, including two pharmacists, two infection control specialists, and an epidemiologist, and is available on request from the corresponding author.

The goal of the survey component of this study was to pilot test the questionnaire in our group of interview participants. Thus, the sample size was driven by the number of people interviewed (20) and the study was not powered for analytic statistical analysis. This paper presents descriptive statistics based on demographic information collected and responses to the open-ended survey question: *Give your best definition of antibiotics*.

### Data quality

In designing our study, we were cognizant of the fact that our study population could have low health literacy and that the very term “antibiotic” might be poorly understood. Therefore, we proactively employed several techniques to facilitate the collection of high quality data. Prior to asking any questions including the word “antibiotic,” we asked participants if they had heard of the term, and if they had, we asked them to define it so that we could assess the accuracy of their understanding. All participants were then provided a definition for antibiotics and given several examples of common antibiotic commercial names used locally. Interview responses to key questions were also cross-checked with responses to six related questions in the structured questionnaire. In the case of discrepancy, the relevant parts of interview transcripts were reanalyzed and categorized based on confidence with the interview response.

Another potential data quality issue was ambiguity regarding colloquial use of the term “doctor.” Participants had a clear understanding of the differences between MBBS doctors and RMPs, and could easily identify providers of each type. However, because in India “doctor” can refer to a wide range of medical professions and expertise, we took particular care to have participants specify what they meant when using this word. The terms MBBS and RMP are used colloquially in these communities, so in times of ambiguity we asked the clarifying question “When you say doctor, do you mean MBBS, RMP, or someone else?”

### Ethical approvals

The Institutional Review Board (IRB) at Medanta the Medicity Hospital, Haryana, India approved this study. An additional independent Ethics Committee at Medanta the Medicity Hospital also granted the study ethics approval. As with all human studies approved by the Institutional Review Board and Ethics Committee at Medanta the Medicity Hospital, a rigorous review and approval process was undertaken with particular consideration for the informed consent process. Our protocol specified that oral consent be obtained instead of written consent. This was because we expected that many participants would have low levels of literacy and we wanted to ensure protection of the rights of all study participants. All participants provided oral consent before any data were collected. A waiver of full review was requested from the Health Sciences IRB at the University of Wisconsin, because the risks for participants in this study were minimal. The Health Sciences IRB granted this study exemption from review. Participants were given 350 Indian Rupees, approximately $5.50 United States Dollars (USD), as compensation for their time. This was deemed an appropriate amount by members of the research team in Haryana.

## Results

### Participants’ characteristics

Twenty community members were recruited from five villages in Haryana. Eighty-five percent of participants were male (17/20). According to survey responses, approximately one-quarter had completed a high school education (6/20, 30%). Two participants were illiterate (10%). Household monthly income (MI) levels ranged from <$30 USD to >$749, with most participants reporting low-middle MI of $65 - $374. Eighty percent (16/20) of participants had children.

Participant responses to six key questions regarding one’s first and second choices for medical care, cessation of antibiotics upon symptom relief in mild and serious illnesses, sharing antibiotics among family, and purchasing antibiotics without a prescription were consistent between interview and survey data 87% of the time.

### Limited healthcare access

MBBS doctors are the only medical providers trained in allopathic medicine and licensed to prescribe antibiotics. However, 85% of participants reported that there was no MBBS doctor in their village (17/20). They had to travel outside of their village to visit an allopathic MBBS doctor. For many, travel costs were a financial burden that made seeking care at allopathic clinics additionally prohibitive.

*“For mild fever, etc., he [RMP] charges 10 or 20 rupees ($0.15 - 0.30 USD). If he were to give an injection, then he charges 30 or 40 rupees [$0.45 - 0.60 USD. … For an MBBS doctor the cost would be] more than this, and travelling to and from would be an additional […approximate total cost of] 1000 rupees ($15 USD).”* - Ninth grade education, MI $150–374

For those living close to another village or city with an MBBS doctor, travel was less of a concern.

*“We go directly to the doctor [MBBS]. He writes a prescription and then we take that to the medical store. […] We have one MBBS doctor here and we go to him. He is a good doctor. […] He is nearby, about 10 min away.”* - Fourth grade education, MI $150–374

Most villages have one or more medical stores and 50% of participants reported going directly to these pharmacies as their first choice for medical care (10/20). Many were highly reliant on the care provided by pharmacists and unlicensed RMPs.

*“I go directly tell my problem to medical store guy, such as I have a cold or a fever, and then I ask him to give me the medication for it”*- Bachelor of Arts, MI > $749

*“In case of mild illness, I buy medicine from the medical store. […] For fever, cold, cough and other smaller problems, I prefer the medical store.”* - Ninth grade education, MI $65–149

*“[I buy medication] from the medical store, and some small medicine we get from our village doctor [RMP. …] If our village doctor is not available, I go to the medical store and say, for example, I have a stomach ache. Please give me medicine for it.”* - Ninth grade education, MI $150–374

Yet, some were concerned about the ability of RMPs to diagnosis and treat conditions correctly.

*“Because he is a quack [RMP], we do not have confidence in him. We are not sure if he knows much about medicines. [… We go to him first, though,] because he is the nearest. Others are located far from here.”* - Tenth grade education, MI $150–374

“*First, I buy medicine from the medical store. [… If I need more care] I will go to a nearby doctor in my village. [What’s his qualification?] Not very qualified, just an RMP.”*- Tenth grade education, MI $375–749

*“I have already been on a medication for many years [participant has a chronic medical condition. …]. I am afraid that if I go to the nearby doctor [RMP, instead of his established MBBS doctor] he might not be able to understand what the problem is. So, usually for a small thing I go directly to a medical store. If the problem has not happened before, then I go to my doctor only [MBBS], no other doctor.”* - Bachelor of Commerce, MI > $749

### Economic factors

Fifteen participants (75%) had a household income of less than $375 per month. The high cost of allopathic medical care was reported by some participants to be prohibitive, driving participants to seek treatment directly from a pharmacy without a doctor’s consultation or prescription.

*“If someone feels something in their body, what do they do? Instead of going to the doctor, because this is a small issue and the doctor will charge a more expensive fee, they will go directly to the medical store. The pharmacist will give them a tablet, etc., as per their understanding. That provides relief up to 75-80% of the time.”* - Tenth grade education, MI $150–374

Patients with mild illness may experience relief after two or three antibiotic doses, which was often perceived as curing the disease. Thus, purchasing a few tablets of a medication, instead of the complete course, was a common practice used to limit medical costs during periods of low income. In the survey, 65% of participants said that they usually or sometimes stop taking antibiotics upon symptom relief in the case of a mild illness (13/20; 55% usually, 10% sometimes). However, the prevalence of this practice was highly dependent on monthly income. It occurred in 85.7% of participants making between $65 and $149 a month, but dropped to 71.4% for those making $150–374, and 50 % for those making over $749. This finding was consistent in the survey participants’ interview data.

*“Sometimes I have less money. [… In these cases] I will buy the important medicine. I will buy fewer pills [not all those recommended] and later on, if required and I have the money, I will buy the remaining.” -* Ninth grade education, MI $65–149

*“If I have less money, then I will buy less medication. Otherwise, I purchase the complete course.” -* Twelfth grade education, MI $150–374

*“It [cost] is not important. What is important is my body.”* - Masters in Business Administration, MI > $749

In the case of a serious illness, participants were more likely to prioritize medical expenses and purchase a full dose of medication. In fact, only 20% reported that they usually or sometimes stop antibiotics early in the case of serious infection.

*“See, the [serious] infection is inside and is not visible. If it was outside, we could have visually seen that it was being cured. Then, the patient gets confidence, so [he will stop treatment in] two or 3 days. If it was inside, then one should take the complete course. [… If it is a milder one] then we can take [the medication] for fewer days.”* - Tenth grade education, MI $150–374

*“If the disease is serious, then it is not good to stop the medicines. The course of the treatment should be completed.”* - Tenth grade education, MI $150–374

### Health education and antibiotic knowledge

Approximately half of the community members reported learning about health topics in school or through a community program (8/17, 52.9%; not assessed in three participants). These classes were typically taken in elementary school and had focused on hand hygiene and general cleanliness.

*“We are told about cleanliness right from class one.”* - Eighth grade education, MI $65–149

*“They used to talk about cleanliness, food habits, […] washing hands before and after having food, cleaning the mouth before going to bed and after getting up in the morning. [… We learned this in] fifth class, as kids are mature by then.”* - Tenth grade education, MI $150–374

Overall, participants had minimal understanding of antibiotics and antibiotic resistance. According to the survey, most participants had heard the term antibiotic before (16/20; 80%), however, none were able to give a complete answer to the open-ended follow-up question: *Give your best definition of antibiotics*. Several responded that antibiotics cure diseases, but did not differentiate between infectious and other types of diseases.

*“Usually we take antibiotics when our immune system does not match with the weather, or anything when we don’t feel good.”* - Bachelor of Commerce, MI > $749

*“Antibiotics are a medicine that the doctor will give in all types of disease.”* - Tenth grade education, MI $375–749

Participants were also largely unaware of the adverse effects of antibiotic misuse and described this medication class as a type of magic bullet that both cures all disease and does no harm. In the survey, less than half of participants said that antibiotics could stop working if taken repeatedly (45%, 9/20).

*“It [an antibiotic] is a medicine one can take in any disease. It will not harm.”* - Tenth grade education, MI $150–374

*“An antibiotic is a drug that will not create any problem in our bodies*.” - Seventh grade education, MI $65–149

*“Antibiotics are drugs that will not cause any reaction [side effect].”* - Eighth grade education, MI $150–374

### Children

The majority of participants (16/20, 80%) had children, with an average number of two (2.19). They were much less willing to take risks regarding their childrens’ health than their own and they were more likely to report taking them first to an allopathic MBBS doctor instead of an RMP or pharmacist.

*“My child takes their medication according to the doctor’s advice only. For the child, I do not take any risks or purchase medication from the pharmacy [without a prescription]. For myself, I purchase directly from the pharmacy. For my child, for any problem, I only go to the doctor.”* - Bachelor of Commerse, MI > $749

No clear pattern appeared regarding how long to treat children in the case of a mild infection. Some participants were adamant about providing a full course of treatment for their children.

*“In the case of children, we are not negligent. Whatever the doctor asks us to do we will do. We are negligent with ourselves, but for ourselves we will complete a full course.”* - Tenth grade education, MI $150–374

*“[For serious disease] I will complete the course. [… Also, for mild disease, I will] complete the course.”* - Twelfth grade education, MI $150–374

Yet, others reported that complete courses were only necessary in the case of serious illness. For mild illness they felt that children, like adults, could stop treatment once they were asymptomatic.

*“I will do the same [for my children as I do for myself]. The doctor gives us a medicine and tells us to administer three doses. If the child is better, then we are to stop taking medicine. There is no use of giving further treatment. [… However, in serious disease] we will continue to give treatment to our children until the doctor tells us to stop.”* - Ninth grade education, MI $150–374

*“[For adults we] stop the medicine earlier, for the sake of not taking it further, because you feel better. We will do the same for kids also […] if their disease is cured.”* - Tenth grade education, MI $150–374

## Discussion

Our findings show that the social factors of income and healthcare access are associated with antibiotic use practices. Antibiotics and the implications of their misuse were poorly understood by the participants. A low baseline level of antibiotic knowledge was consistent across a range of educational attainment and in-school health learning experiences.

In the survey, over half of participants reported that it is appropriate to stop antibiotics upon symptom relief (65%). While the study populations are not comparable, it is important to note that 65% is much higher than the 37% result found in a recent WHO survey of antibiotic use in India [35]. The WHO’s sample was more educated (69% received some higher education) and more urban (9% lived in rural areas). Across the WHO study, educated participants and those in urban areas were more likely to report that a complete dose of antibiotics should be taken. As in our study, the WHO found that low-income populations were especially prone to support early termination of antibiotic treatment. This may be due in part to an interaction between income and antibiotic knowledge. If patients come from a low income group and are unaware of the dangers of prematurely stopping treatment, it is both the logical and economical decision to stop taking antibiotics once symptoms resolve.

Our results are in agreement with previous studies investigating the pharmacist-patient relationship in India, which also reported low income as a driver of antibiotic misuse. In a recent study of pharmacists in New Delhi, pharmacists cited requests from poorer patients as a reason for selling shortened antibiotic courses [21]. As in our study, pharmacists considered this a type of charity work for the community. A study of patients in the southern state of Kerala found that purchasing antibiotics without a prescription was least common among high income and well educated patients [22].

In our study, the financial burden of expensive allopathic medical visits also led many study participants to purchase antibiotics directly from a pharmacist or RMP without a prescription. Pharmacists were viewed by many participants as knowledgeable members of the healthcare community who provide trustworthy and accurate clinically advice. However, many people dispensing medications at pharmacies have no formal education in clinical pharmacy [27]. These “pharmacists” often dispense antibiotics without prescriptions based on informal training from co-workers and past experience. Similarly, RMPs have no formal medical education and train through experience. They are not legally sanctioned to practice medicine, however, the high efficacy of antibiotics against infections has led unlicensed practitioners to dispense antibiotics directly from their clinics. Future studies should investigate antibiotic dispensing practices among RMPs, as well as licensed and unlicensed pharmacists.

Because 85% of participants had no access to a licensed allopathic MBBS doctor in their own village, travel costs were another reported factor in the decision whether or not to seek care directly from a pharmacist. Villages in India are defined by population size, not urbanicity. Urban village populations are less geographically isolated and have easier access to cities than rural villages for all services, including healthcare. The urban villages we studied had been surrounded over the past decade by the rapidly expanding city of Gurgaon, a major medical hub with hundreds of practicing MBBS doctors. All rural and urban villages in our study were within a 90-min radius from Gurgaon. There is a need for future studies to examine the relationship between travel costs, healthcare utilization, and antibiotic misuse in rural villages located further from urban centers.

Our findings among parents, with regards to healthcare seeking for their children, is promising. Parents relied more on allopathic doctors and less on direct utilization of pharmacists for their children’s care. This practice, seemingly related to parents’ intrinsic hesitancy to purchase antibiotics without a prescription for their children, should be investigated in future studies, as it could be harnessed to reduce this practice in the adult population as well.

The primary limitation in this study is the potential lack of response authenticity. Purchasing antibiotics without a prescription is illegal in India [20], thus, participants may have underreported this and other socially undesirable activities. We sought to minimize this effect by conducting the interview in participants’ home villages and having buy-in from local pharmacies. In many cases, behaviors that lead to antibiotic resistance were the norm in the communities where we interviewed. This finding was consistent with other studies of antibiotic misuse across India [22, 36]. Therefore, we believe that participants perceived less stigma associated with these behaviors than we initially expected. Ultimately, 60% of participants reported taking antibiotics not prescribed by a licensed allopathic doctor.

Another limitation is the study’s generalizability. Ninety percent of study participants were literate and over one-quarter had completed a high school degree. Antibiotic practices among this more educated cohort may not represent findings in the larger Haryana population. Furthermore, as a country, India has a very heterogeneous population and healthcare profile. While our findings regarding the effect of income on antibiotic purchasing and use are consistent with prior studies from throughout the country [21, 22], it is unlikely that all our results will be transferable to all contexts and settings across India.

## Conclusions

Our study found that many community members in Haryana villages have limited healthcare access, minimal understanding of proper antibiotic practices, and live in poor economic conditions. The prevalent misuse of antibiotics among these community members reinforces the importance of conducting research to develop effective strategies for stemming the tide of antibiotic resistance in India’s villages.

## Abbreviations

BAMS: Bachelor in Ayurvedic Medicine and Surgery
BHMS: Bachelor in Homoeopathic Medicine and Surgery
BUMS: Bachelor in Unani Medicine and Surgery
MBBS: Bachelor of Medicine, Bachelor of Surgery
MDRO: Multidrug resistant organism
MI: Monthly income
RMP: Rural medical practitioner
USD: United States Dollars
